# Association of Length of Stay, Recovery Rate, and Therapy Time per Day With Functional Outcomes After Hip Fracture Surgery

**DOI:** 10.1001/jamanetworkopen.2019.19672

**Published:** 2020-01-24

**Authors:** Alison M. Cogan, Jennifer A. Weaver, Matt McHarg, Natalie E. Leland, Leslie Davidson, Trudy Mallinson

**Affiliations:** 1 Department of Physical Medicine and Rehabilitation, Washington DC VA Medical Center, Washington, DC; 2 School of Medicine and Health Sciences, Clinical Research and Leadership, The George Washington University, Washington, DC; 3 School of Medicine and Health Sciences, The George Washington University, Washington, DC; 4 Department of Occupational Therapy, School of Health and Rehabilitation Sciences, University of Pittsburgh, Pittsburgh, Pennsylvania

## Abstract

**Importance:**

Medicare is shifting from payment for postacute care services based on the volume provided to payment based on value as determined by patient characteristics and functional outcomes. Matching therapy time and length of stay (LOS) to patient needs will be critical to optimize functional outcomes and manage costs.

**Objective:**

To investigate the association among therapy time, LOS, and functional outcomes for patients following hip fracture surgery.

**Design, Setting, and Participants:**

This retrospective cohort study analyzed data on patients from 4 inpatient rehabilitation facilities and 7 skilled nursing facilities in the eastern and midwestern United States. Participants were patients aged 65 years or older who received inpatient rehabilitation services for hip fracture and had Medicare fee-for-service as their primary payer. Data were collected from 2005 to 2010. Analysis was conducted from November 2018 to June 2019.

**Exposure:**

Therapy minutes per LOS day.

**Main Outcomes and Measures:**

Functional Independence Measure mobility and self-care measures at discharge. Patients were categorized into 9 recovery groups based on low, medium, or high therapy minutes per LOS day and low, medium, or high rate of functional gain per day.

**Results:**

A total of 150 patients (101 [67.3%] female; 148 [98.6%] white; mean [SD] age, 82.0 [7.3] years) met inclusion criteria. Participants in all gain and therapy minutes per LOS day trajectories were similar in function at rehabilitation admission (mean [SD] mobility, 16.2 [3.2]; *F*_8,141_ = 1.26; *P* = .27) but differed significantly at discharge (mean [SD] mobility, 23.9 [5.2]; *F*_8,141_ = 14.34; *P* < .001). High-gain patients achieved mobility independence by discharge; low-gain patients needed assistance on nearly all mobility tasks. Medium-gain patients with a mean LOS of 27 days were independent in mobility at discharge; those with a mean LOS less than 21 days needed supervision with toilet transfers and were dependent with stairs. Length of stay and functional gain rate explained much of the variance in mobility and self-care scores at discharge. Although medium– and high–therapy minutes per LOS day groups were statistically significant in the regression model (β = 6.99; *P* = .001; and β = 11.46; *P* = .007, respectively), they explained only 1% of the variance in discharge outcome. Marginal means suggest that medium-gain patients with shorter LOS would have achieved mobility independence if LOS had been extended.

**Conclusions and Relevance:**

In this study, rate of recovery and LOS in skilled nursing and inpatient rehabilitation facilities were associated with mobility and self-care outcomes at discharge following hip fracture surgery, particularly for medium-gain patients. Therapy time per day explained only 1% of the variance in discharge outcome. Discharging medium-gain patients before 21 days LOS may transfer burden of care to family and caregivers, home health, and outpatient services.

## Key Points

**Question:**

Is there an association between therapy minutes per day and mobility and self-care outcomes for patients receiving rehabilitation services in postacute care facilities after undergoing hip fracture surgery?

**Findings:**

This cohort study of 150 participants found that rate of recovery and length of stay were associated with discharge functional outcomes. Therapy minutes per length of stay day explained only 1% of variance in discharge mobility and self-care outcomes.

**Meaning:**

These findings suggest that for patients who improve at a medium rate, shorter length of stay (<21 days) may transfer additional burden of care to family caregivers, home health agencies, and outpatient services.

## Teaser

This cohort study investigates the association among therapy time, length of stay, and functional outcomes for patients aged 65 years or older recovering from hip fracture surgery in postacute care facilities.

## Introduction

Medicare payment models for postacute care (PAC) services are shifting from a model based on volume of services provided, such as amount of therapy (occupational, physical, and speech/language) or length of stay (LOS), to one of value based on patients’ clinical characteristics. Historically, the amount of therapy provided in PAC facilities has been driven by reimbursement requirements rather than patient need as determined by clinical teams. This change from volume to value is intended to encourage health care professionals to optimize functional gains for patients while minimizing costs and lessen the financial incentives to provide more therapy than necessary.^1^ Under a value-based system, rehabilitation teams will need to make evidence-informed decisions about the amount of therapy per day and LOS to maximize patients’ functional improvement.

Aronow et al^2^ found that patients recovered from the time of joint replacement surgery for hip fracture to PAC rehabilitation admission along 3 distinct trajectories that were associated with outcomes at an 8-month follow-up interview. Prior research, however, shows little variation in rehabilitation therapy volume after hip fracture surgery,^3,4,5^ which makes it difficult to identify an association between therapy dosage and patient outcomes. For instance, differences in self-care measures at discharge between patients with hip fracture at either inpatient rehabilitation facilities (IRFs) or skilled nursing facilities (SNFs) became nonsignificant when controlling for LOS, suggesting that natural recovery accounted for some of the improvement.^6^

Older adults receiving PAC rehabilitation services after hip fracture surgery often present with complex medical needs that influence their recovery trajectories.^7^ Common geriatric conditions such as pressure ulcers, incontinence, falls, functional decline, and delirium^8^ affect a person’s ability to participate in rehabilitation interventions, thereby limiting the benefits of these skilled services. Patients who do not sufficiently recover mobility and self-care ability after hip fracture are at increased risk for hospital readmissions.^9^

Because rehabilitation time in PAC has been driven by volume-based payment reimbursement rather than patient characteristics and needs, evidence to support the optimal amount of therapy time per day is lacking. To better understand how rehabilitation teams can make decisions about therapy dosage for PAC patients, the purpose of this study is to investigate the association between therapy minutes per LOS day (TMLD) and discharge mobility and self-care outcomes for patients who receive rehabilitation services in PAC facilities after hip fracture surgery.

## Methods

### Study Design

This article reports a retrospective analysis of data from a multicenter prospective observational cohort study from 2005 to 2010, with analysis conducted from November 2018 to June 2019. Details about recruitment and data collection methods are described elsewhere.^6,10^ The parent study was approved by the institutional review boards of Northwestern University and each of the participating facilities. All participants completed a written informed consent and had undergone hip fracture surgery at time of study enrollment. The present study was considered exempt from review by the George Washington University institutional review board because it posed less than minimal risk to participants. In addition to demographic and health condition data collected for the parent study, setting-specific functional assessments (Minimum Data Set 2.0, Functional Independence Measure [FIM], and the Outcome and Assessment Information Set) were collected on all participants, regardless of rehabilitation setting, at both PAC admission and discharge. Having common assessment data allows for direct comparison of participants across settings (ie, inpatient rehabilitation, skilled nursing, or home health), which reduces some of the selection bias. This report follows the Strengthening the Reporting of Observational Studies in Epidemiology (STROBE) reporting guideline.^11^

### Facilities

Data from 4 IRFs and 7 SNFs are included. All facilities were in the eastern and midwestern United States.

### Participant Selection and Characteristics

Participants were selected if they met the following eligibility criteria: (1) admission to PAC following surgery for hip fracture, (2) age 65 years or older, (3) received rehabilitation therapy services, (4) primary payer source was documented as Medicare fee-for-service, and (5) had complete admission and discharge records. Information on demographic characteristics (sex, age, and race), social supports (marital status, living situation), and specific impairments and comorbidities that may influence a person’s ability to participate in or benefit from therapy were obtained. Total number of comorbidities was calculated from the number of conditions reported in the medical record (up to a total of 10 conditions). We also looked at geriatric conditions^8^ known to influence therapy participation, including obesity, visual impairment, diabetes, cognitive impairment, dementia,^12^ depression, and falls during PAC stay.

### Therapy Measures and Functional Status

The primary outcome was FIM mobility and self-care scores. Research nurses scored participants’ most dependent functional level during the first 48 hours of PAC admission and last 48 hours before discharge on the FIM rating scale from 1 (total dependence) to 7 (independent). In order of difficulty, the 7 mobility items were toilet transfers (least challenging), bed transfers, wheelchair, shower transfer, tub transfer, walking, and climbing stairs (most challenging).^10^ In order of difficulty, the 6 self-care items were eating (least challenging), grooming, upper-extremity dressing, toileting, bathing, and lower-extremity dressing (most challenging).^10^

Items assessed by the FIM scale were transformed into equal-interval Rasch measures for each domain using Winsteps software version 3.6.9; details are reported elsewhere.^6^ To ensure interpretability, the Rasch measures were transformed to match the expected raw score range for mobility (7-49) and self-care (6-42).

### Independent Variables: Gain Rate per LOS Day and TMLD

Gain rate per LOS day was calculated separately for mobility and self-care as the difference between discharge and admission FIM scores divided by LOS days. We calculated TMLD as the total therapy minutes (occupational therapy, physical therapy, speech language pathology, psychology, respiratory therapy, and any additional therapies) divided by LOS days (eFigure 1 in the Supplement). Most therapy minutes were occupational and physical therapy, which is typical for this patient population. The content of therapy sessions was not available. Because participants may not have received therapy on all LOS days, we also report the number of days without occupational and physical therapy (eTable 1 and eTable 2 in the Supplement).

### Statistical Analysis

All analyses were conducted using Stata software version 15.1 (StataCorp LLC). We distinguished groups by low, medium, and high TMLD. Our goal was to have balanced groups defined by clinically acceptable definitions of low, medium, and high minutes per LOS day. Cut points for TMLD were as follows: low, less than 80 min/LOS day (<560 min/wk); medium, 80-130 min/LOS day (560-910 min/wk); and high, more than 130 min/LOS day (>910 min/wk). The cut points approximate different PAC reimbursement levels; the threshold between low and medium TMLD is close to that between the high and very high SNF resource utilization groups (500 min/wk) and high TMLD reflects the so-called 3-hour rule for IRF (900 min/wk).^13,14^

Gain per LOS day was also divided into balanced low, medium, and high groups (calculated separately for mobility and self-care). We cross-mapped the gain and TMLD groups to create 9 recovery trajectories (eg, low gain, low TMLD; low gain, medium TMLD) separately for mobility and self-care. Median mobility and self-care measures at admission and discharge and net change for each gain and TMLD trajectory were calculated (eTable 1 and eTable 2 in the Supplement). We used 1-way analysis of variance and Tukey honestly significant difference test to assess differences in admission and discharge mobility and self-care measures across the 9 trajectory groups. Frequency of geriatric conditions within each of the 9 trajectories was examined. Stepwise linear regressions were used to explore the contributions of admission measure, LOS, TMLD, gain group, and geriatric conditions to discharge mobility and self-care measures. The Akaike information criterion and Bayes information criterion were calculated for each iteration to guide identification of the best-fitting model. Marginal means were calculated from the final regression model to project mobility and self-care outcomes at different LOS. Two-sample *t* tests were used to compare primary outcomes in IRFs and SNFs. Statistical significance was set at 2-tailed *P* < .05.

## Results

Of 150 participants who met the inclusion criteria, 101 (67.3%) were female and 148 (98.6%) were white; patients had a mean (SD) age of 82.0 (7.3) years and were evenly distributed between IRF and SNF settings (52.7% and 47.3%, respectively; Table 1). The most commonly reported geriatric conditions were bladder incontinence (52.0%), impaired decision-making (42.7%), and visual impairment (22.7%) (eTable 3 and eTable 4 in the Supplement). Eighty-eight participants (58.7%) had 8 to 10 reported comorbidities. One participant was discharged back to an acute care facility and was missing discharge FIM measures; this patient was excluded from the analysis.

**Table 1.** Patient Demographic Characteristics

Variable	No. (%)		
	IRF (n = 79)	SNF (n = 71)	Total (N = 150)
Female	48 (67.6)	53 (74.6)	101 (67.3)
Age, mean (SD), y	80.73 (6.95)	83.36 (7.53)	82.0 (7.3)
Race			
White	78 (98.7)	70 (98.6)	148 (98.6)
Other	1 (1.3)	1 (1.4)	2 (1.4)
Marital status			
Widowed	33 (41.8)	24 (33.8)	72 (48.0)
Married or partnered	33 (41.8)	39 (54.9)	57 (38.0)
Divorced	5 (6.3)	1 (1.4)	11 (7.3)
Single	6 (7.6)	5 (7.0)	6 (4.0)
Unknown	0	1 (1.4)	1 (0.7)
Lives alone	33 (41.8)	36 (50.7)	69 (46.0)
Discharge to home	56 (70.9)	53 (73.2)	108 (72.0)
Geriatric conditions^a^			
Bladder incontinence	42 (53.2)	36 (50.7)	78 (52.0)
Impaired decision-making	22 (27.8)	42 (59.2)	64 (42.7)
Visual impairment	21 (26.6)	13 (18.3)	34 (22.7)
Diabetes	18 (22.8)	14 (19.7)	32 (21.3)
Bowel incontinence	14 (17.7)	12 (16.9)	26 (17.3)
Depression	12 (15.2)	10 (14.1)	22 (14.7)
Obesity	8 (10.1)	9 (12.7)	17 (11.3)
Falls	8 (10.1)	1 (1.4)	9 (6.0)
Dementia^b^	0	3 (4.2)	3 (2.0)
Memory impairment			
Short-term	10 (12.7)	25 (35.2)	35 (23.3)
Long-term	3 (3.8)	8 (11.3)	11 (7.3)
Total reported comorbidities, No.^c^			
0-3	5 (6.3)	7 (9.9)	12 (8.0)
4-7	12 (15.2)	38 (53.5)	50 (33.3)
8-10	62 (78.5)	26 (36.6)	88 (58.7)

Abbreviations: IRF, inpatient rehabilitation facility; SNF, skilled nursing facility.

^a^ Geriatric conditions derived from geriatric syndrome and risk factors as described by Inouye et al.^8^

^b^ Dementia calculated from Cognitive Performance Scale score per algorithm described by van der Steen et al.^12^

^c^ Reflects the count of any recorded comorbidities, up to 10 reported on data collection forms.

Therapy minutes per LOS day ranged from less than 40 to more than 200. Median LOS varied from 10.5 to 35.0 days (eFigure 2 and eFigure 3 in the Supplement). Participants were classified as receiving low (44 patients), medium (52 patients), or high (54 patients) TMLD. Most participants in the low-TMLD group were treated in SNF settings and all but 1 in the high-TMLD group were treated in IRF settings. In the medium-TMLD group, participants were split between IRF (27 patients) and SNF (25 patients). The low-TMLD group had more nontherapy days than other groups but also had longer LOS (eTable 1 and eTable 2 in the Supplement).

### Mobility

Mobility gains were categorized as low (<0.25 units/LOS day; 49 participants), medium (0.25-0.50 units/LOS day; 51 participants), and high (>0.50 units/LOS day; 50 participants). Functional mobility status at admission was not significantly different across the 9 trajectory groups (mean [SD] mobility, 16.2 [3.2]; *F*_8,141_ = 1.26; *P* = .27) but differed significantly at discharge (mean [SD] mobility, 23.9 [5.2]; *F*_8,141_ = 14.34; *P* < .001).

**Figure 1.**
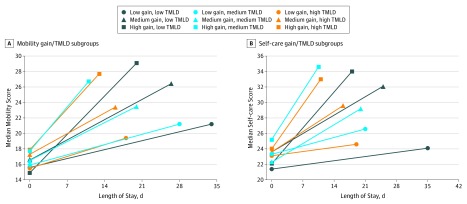
Hip Fracture Recovery Trajectories by Gain and Therapy Minutes per Length of Stay Day (TMLD) Subgroup A, Mobility measures 26 or greater indicate minimum assistance for stairs and modified independent or supervised status on all other items. Measures 22 or greater but less than 26 indicate supervision for toilet transfers, maximum assistance for stairs, and minimum assistance to supervised status for other items. Measures less than 22 indicate minimum assistance for toilet transfers and moderate to maximum assistance for other items. B, Measures greater than 30 indicate supervised to independent status on all items. Measures 27 to 30 indicate need for minimum assistance for lower-extremity dressing, bathing, and toileting. Measures less than 27 indicate moderate to maximum assistance for lower-extremity dressing and bathing, minimum to moderate assistance for toileting, and supervision for upper-extremity dressing.

Figure 1A presents the mobility recovery trajectories for each of the 9 groups, plotting admission and discharge measures against LOS days; the slope of the line connecting these 2 points represents the median gain per LOS day for each group. Mobility measures 26 or greater indicate modified assistance or independence on all items except stairs, suggesting participants could be safely discharged to the community with respect to mobility. Measures 22 or greater but less than 26 indicate need for minimum assistance with most items, supervision with toilet transfers, and maximum assistance with stairs, signifying the need for support after discharge with regard to mobility. Measures less than 22 denote need for moderate to maximum assistance with most items, indicating participants would need full-time assistance after discharge with regard to mobility. Participants in the low-gain trajectories had median discharge mobility measures indicative of needing high levels of assistance regardless of the LOS or TMLD. Participants in all high-gain trajectories had median discharge measures indicating minimal assistance needed only for climbing stairs, also regardless of TMLD. However, participants with high gain and low TMLD had a LOS 7 days longer than those with high gain and high TMLD. Although their functional status at admission was not significantly different, participants in the low-gain trajectories never reached the same level of mobility as those in the high-gain groups, regardless of TMLD or LOS. Mobility outcome was not significantly different by facility type. Disorders of the nervous system (eg, Parkinson disease, multiple sclerosis) were not associated with gain group, TMLD, or discharge outcomes.

**Figure 2.**
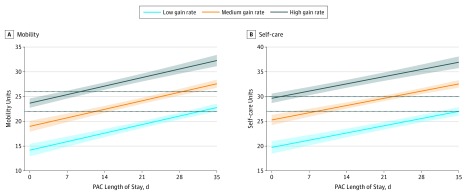
Hip Fracture Recovery Marginal Means Model Marginal means for mobility (A) and self-care (B) were calculated from the regression model controlling for mobility score at admission, therapy minutes per length of stay day, length of stay, having 3 or more geriatric conditions, and interaction between gain rate group and therapy minutes per length of stay day group. Dashed lines show the functional independence thresholds at 22 and 26 units (A) and at 27 and 30 units (B), and shaded areas show 95% confidence intervals. PAC indicates postacute care.

Table 2 shows each iteration of the stepwise linear regression model. The final regression model confirmed that, after controlling for admission mobility measure, TMLD, the interaction between gain and TMLD, LOS, and having 3 or more geriatric conditions, belonging to the medium-gain group (β = 6.99, *P* = .001) or the high-gain group (β = 11.46, *P* = .007) was associated with better discharge mobility compared with the low-gain group. Although medium- and high-TMLD groups were statistically significantly different from low TMLD in the final model, the contribution of TMLD to the overall model was modest as indicated by the Akaike information criterion and Bayes information criterion, explaining only 1% of the variance in the discharge outcome. Length of stay was significantly associated with discharge mobility measure and explained much of the variance in the overall model. Medium-gain patients with a mean LOS of 27 days were independent in mobility at discharge; those with a mean LOS less than 21 days needed supervision with toilet transfers and were dependent with stairs. Marginal means models suggest that longer LOS is associated with improved mobility outcome, especially for participants in the medium-gain group, regardless of TMLD (Figure 2A). Faster gain was associated with better discharge mobility function. Within each gain group, LOS was the most important factor associated with functional status at discharge, while TMLD was only weakly associated.

**Table 2.** Stepwise Regression Model for Mobility Discharge Score Including All Variables

Variable	Adjusted *R*^2^	AIC	BIC	β (SE)^a^										
				Admission Mobility Score	Mobility Gain Group		TMLD Group		Gain and TMLD				≥3 Geriatric Conditions	LOS
					Medium	High	Medium	High	Medium and Medium	High and Medium	Medium and High	High and High		
Admission mobility score	0.51	852.83	858.85	1.00 (0.03)^b^										
Mobility gain group	0.61	781.19	793.23	0.76 (0.08)^b^	3.95 (0.65)^b^	6.50 (0.67)^b^								
TMLD group	0.62	776.52	794.58	0.76 (0.08)^b^	4.21 (0.65)^b^	7.14 (0.69)^b^	–1.43 (0.67)^c^	–1.94 (0.67)^d^						
Gain and TMLD interaction	0.63	776.24	806.35	0.75 (0.08)^b^	5.85 (1.03)^b^	7.61 (1.55)^b^	0.62 (1.08)	–1.55 (1.08)	–3.33 (1.53)^c^	–2.50 (1.89)^c^	–2.01 (1.53)	0.40 (1.88)		
≥3 Geriatric conditions	0.64	773.47	806.59	0.70 (0.08)^b^	5.66 (1.02)^b^	7.44 (1.53)^b^	0.17 (1.09)	–1.71 (1.07)	–3.12 (1.51)^c^	–2.41 (1.87)	–1.83 (1.52)	0.31 (1.85)	–1.26 (0.59)^c^	
LOS	0.82	671.66	707.79	0.92 (0.06)^b^	6.99 (0.73)^b^	11.46 (1.14)^b^	2.21 (0.79)^d^	2.15 (0.83)^c^	–3.01 (1.07)^d^	–3.26 (1.33)^c^	–3.17 (1.08)^d^	–2.32 (1.33)	–0.91 (0.42)^c^	0.25 (0.02)^b^

Abbreviations: AIC, Akaike information criterion; BIC, Bayes information criterion; LOS, length of stay; TMLD, therapy minutes per LOS day.

^a^ Regression coefficients are provided for each model relative to low-gain, low-intensity groups. Gain group remained significant in all models. Therapy intensity was not significant after interacting with gain group, although medium-intensity, medium-gain and medium-intensity, high-gain groups were significant. Neither intensity nor geriatric conditions significantly improved the amount of model variance explained. Length of stay dramatically increased the proportion of variance explained, and all therapy group coefficients became significant, indicating that LOS explains most of the difference in mobility discharge measure among the groups. Smaller numbers for both AIC and BIC indicate a better-fitting model.

^b^ Statistically significant at *P* ≤ .001.

^c^ Statistically significant at *P* ≤ .05.

^d^ Statistically significant at *P* ≤ .01.

### Self-care

Participants were categorized as having low (<0.20 unit/LOS day; 40 patients), medium (0.20-0.50 unit/LOS day; 68 patients), and high (>0.50 units/LOS day; 42 patients) self-care gain per day. Most fell into the same gain and TMLD trajectory for self-care as for mobility. One-way analysis of variance results indicated significant differences in admission self-care measures (mean [SD] self-care, 22.5 [3.4]; *F*_8,150_ = 2.70; *P* = .01); post hoc Tukey honestly significant difference analysis indicated no significant differences between the 9 groups. At discharge, there were significant differences among trajectory groups on self-care measures (mean [SD], 29.2 [5.3]; *F*_8,141_ = 14.04; *P* < .001). There was no significant difference in self-care outcome by facility type.

Figure 1B presents the recovery trajectories for each of the 9 gain and TMLD self-care trajectories by plotting admission and discharge self-care measures against LOS days. Self-care measures 30 or greater denote supervision or independence on all items, indicating these participants could be safely discharged to the community with respect to self-care. Measures 27 to 30 indicate need for minimum assistance with the more challenging items (lower-extremity dressing, bathing, and toileting), implying participants will need some support after discharge with regard to self-care. Measures less than 27 indicate need for moderate to maximum assistance with lower-extremity dressing and bathing, minimum to moderate assistance with toileting, and supervision for upper-extremity dressing, signaling participants would need skilled assistance for self-care after discharge. As with mobility outcomes, level of independence with self-care tasks indicated by the discharge measure was different for participants in low-, medium-, and high-gain groups. Participants in the low-gain groups had discharge measures that indicated moderate to maximum assistance required for lower-extremity dressing, bathing, and toileting. Participants in the high-gain groups had discharge measures that indicated they could perform lower-extremity dressing with supervision and all other self-care tasks independently.

Table 3 shows the iterations of the stepwise linear regression model for self-care discharge outcome. The final model shows that, when controlling for admission self-care measure, TMLD, interaction between TMLD and gain rate, presence of 3 or more geriatric conditions, and LOS, the medium- and high-gain groups were associated with higher discharge self-care measures relative to the low gain group (β = 8.39; *P* = .001; and β = 12.38; *P* = .001, respectively) (Table 3). The difference between TMLD groups was statistically significant, but TMLD did not improve the explanatory value of the model as measured by the adjusted *R*^2^, Akaike information criterion, and Bayes information criterion. Marginal means models (Figure 2B) suggest that gain group and LOS were most strongly associated with discharge self-care measure, regardless of TMLD.

**Table 3.** Stepwise Regression Model for Self-care Discharge Score Including All Variables

Variable	Adjusted *R*^2^	AIC	BIC	β (SE)^a^										
				Admission Self-care Score	Self-care Gain Group		TMLD Group		Gain and TMLD				≥3 Geriatric Conditions	LOS
					Medium	High	Medium	High	Medium and Medium	High and Medium	Medium and High	High and High		
Admission self-care score	0.55	856.02	862.04	0.93 (0.02)^b^										
Self-care gain group	0.66	769.49	781.54	0.83 (0.08)^b^	5.15 (0.62)^b^	7.46 (0.70)^b^								
TMLD group	0.69	760.61	778.67	0.87 (0.08)^b^	5.22 (0.60)^b^	7.80 (0.69)^b^	–0.87 (0.64)	–2.23 (0.64)^b^						
Gain and intensity interaction	0.69	762.05	792.16	0.86 (0.08)^b^	7.10 (0.96)^b^	8.85 (1.52)^b^	0.88 (1.21)	–0.28 (1.11)	–2.92 (1.49)	–1.60 (1.92)^c^	–3.17 (1.41)^c^	1.95 (1.85)		
≥3 Geriatric conditions	0.69	763.21	796.33	0.83 (0.08)^b^	7.05 (0.96)^b^	8.81 (1.52)^b^	0.81 (1.21)	–0.22 (1.11)	–2.97 (1.49)^c^	–1.67 (1.92)	–3.23 (1.42)^c^	–2.18 (1.87)	–0.54 (0.60)	
LOS	0.82	679.41	715.53	0.89 (0.06)^b^	8.39 (0.74)^b^	12.38 (1.2)^b^	3.29 (0.95)^b^	3.04 (0.89)^b^	–3.94 (1.13)^b^	–2.87 (1.45)^c^	–4.17 (1.07)^b^	–4.08 (1.42)^d^	–0.35 (0.46)	0.21 (0.02)^b^

Abbreviations: AIC, Akaike information criterion; BIC, Bayes information criterion; LOS, length of stay; TMLD, therapy minutes per LOS day.

^a^ Regression coefficients are provided for each model relative to low-gain, low-intensity groups. Gain group remained significant in all models. Therapy intensity remained significant after interacting with gain group, as were medium-intensity, medium-gain and medium-intensity, high-gain groups. Neither intensity nor geriatric conditions significantly improved the amount of model variance explained. Length of stay dramatically increased the proportion of variance explained, and all therapy group coefficients became significant (except geriatric conditions), indicating that LOS explained most of the difference in self-care discharge measure among the groups. Smaller numbers for both AIC and BIC indicate a better-fitting model.

^b^ Statistically significant at *P* ≤ .001.

^c^ Statistically significant at *P* ≤ .05.

^d^ Statistically significant at *P* ≤ .01.

## Discussion

Our data show that mobility and self-care outcomes for participants receiving rehabilitation after hip fracture surgery were associated with LOS and rate of recovery; TMLD explained very little of the variance in outcomes. Similar to Aronow and colleagues,^2^ we identified 3 distinct gain rate groups. The total minutes of therapy patients received and LOS are consistent with other studies^15^ and appeared to be largely determined by the Medicare payment models for IRF and SNF settings.^3,4,5^ These reimbursement-driven therapy volumes make it challenging to understand the dose-response association because TMLD is determined by payment models rather than clinical judgment. We were able to compare participants in IRF and SNF facilities who received the same amount of therapy per LOS day because participants were assessed using the same tools and found no difference in outcomes by setting. Our identification of different recovery trajectories following surgery after hip fracture may support adjustments to prescribed therapy dosage and facility LOS.

With the forthcoming shift to value-based PAC reimbursement,^1^ rehabilitation professionals may have greater flexibility to match both LOS and therapy time to patient needs. However, there is a lack of evidence-informed guidelines describing anticipated patient recovery trajectories at differing amounts of therapy and lengths of stay to support such decisions. It is important for the care team to identify a patient’s recovery trajectory early enough in the episode of care to promote the best possible outcomes and allocate resources efficiently. Plotting a patient’s progress in the first week after PAC admission against the recovery trajectory graphs in Figure 1 may help the team decide how to best adjust the care plan to facilitate functional independence and anticipate necessary external supports at discharge. For example, participants in our sample who showed low gains with medium or high TMLD may have achieved greater functional independence at discharge with less therapy over a longer LOS.

Our findings suggest that some patients, particularly those in the medium-gain groups, may not reach their full recovery potential if LOS is less than 21 days. An additional week of recovery with less therapy time may be more beneficial for achieving greater independence and requiring less physical assistance once at home. Such decisions may have an important impact on transition plans; appropriate discharge support following discharge could also be enhanced.^16^ This information could also benefit family members and caregivers by better anticipating the amount of assistance with self-care and mobility tasks the patient is likely to need upon PAC discharge after hip fracture surgery.

Future research should replicate our findings in a larger sample and focus on identifying patients who are likely to benefit from more vs less therapy time to make optimal recovery after hip fracture surgery. Studies on the costs of PAC rehabilitation and long-term outcomes are also needed. In addition, facilities may consider implementing programs to address modifiable geriatric conditions that limit therapy participation and functional independence, such as weight management and pelvic floor rehabilitation. Medicare reimbursement for such approaches may incentivize program development.^8^

### Limitations

This pilot study has some limitations. We used secondary data analysis with a relatively small sample. Replicating this study with a larger sample could inform the development of reliable clinical guidelines and decision-making tools. We do not have data on content of therapy activities or the reasons why a participant may not have received therapy on a given day (eg, weekend, resource utilization group, medical complications, refusal to participate). Therapy time alone does not indicate how much is accomplished within a treatment session. Data reflect regional US practice and represent a racially homogenous sample. Not all patients with hip fracture in all participating facilities were captured in the parent study. Data were collected from 2005 to 2010 and may not reflect current practice patterns. However, PAC settings have not substantively changed and SNF Medicare reimbursement has only recently changed.

Functional status was measured only at admission and discharge; therefore, we cannot report whether the rate of improvement is constant across the LOS or varies over the course of PAC stay. Accurately describing pace of recovery is important if such graphics as we present are to be used to inform clinical decision-making with caregivers and patients. Factors for which we did not have data, such as prior level of function, severity of comorbid conditions, or protective factors, may have influenced patients’ responsiveness to therapy.^7^ Therapy minutes per LOS day may be more strongly associated with improvement in domains that are not captured by FIM, such as quality of life.

## Conclusions

In this study, rate of gain and LOS were more strongly associated with mobility and self-care functional outcomes at discharge than TMLD for participants receiving rehabilitation services in PAC settings after hip fracture surgery. For participants in medium-gain groups, LOS less than 21 days may have transferred additional burden of care to family caregivers, home health agencies, and outpatient services. In the context of the transition from volume-based to value-based reimbursement under Medicare, evidence-informed guidelines are needed to identify a patient’s recovery trajectory early in the PAC stay so that rehabilitation teams can make patient-driven decisions about LOS and therapy time.

## Notes

Supplement. **eTable 1.** Hip Fracture Unadjusted Mobility Status at Admission and Discharge, Overall Change, Change per LOS Day, and Length of Stay **eTable 2.** Hip Fracture Unadjusted Self-care Status at Admission and Discharge, Overall Change, Change per LOS Day, and Length of Stay **eFigure 1.** Definition of Therapy Minutes per LOS Day **eFigure 2.** Mobility Gain per Day by TMLD, Inpatient Rehabilitation and Skilled Nursing Facility Comparison **eFigure 3.** Self-care Gain per Day by TMLD, Inpatient Rehabilitation and Skilled Nursing Facility Comparison **eTable 3.** Geriatric Conditions by Hip Fracture TMLD/Gain Groups: Mobility **eTable 4.** Geriatric Conditions by Hip Fracture TMLD/Gain Groups: Self-care
