# Supplementary material for: Association of Length of Stay, Recovery Rate, and Therapy Time per Day With Functional Outcomes After Hip Fracture Surgery
Source: JAMA Netw Open. 2020 Jan 24;3(1):e1919672. doi: 10.1001/jamanetworkopen.2019.19672 (PMC6991278; doi:10.1001/jamanetworkopen.2019.19672)
Supplement: Supplement. — eTable 1. Hip Fracture Unadjusted Mobility Status at Admission and Discharge, Overall Change, Change per LOS Day, and Length of Stay eTable 2. Hip Fracture Unadjusted Self-care Status at Admission and Discharge, Overall Change, Change per LOS Day, and Length of Stay eFigure 1. Definition of Therapy Minutes per LOS Day eFigure 2. Mobility Gain per Day by TMLD, Inpatient Rehabilitation and Skilled Nursing Facility Comparison eFigure 3. Self-care Gain per Day by TMLD, Inpatient Rehabilitation and Skilled Nursing Facility Comparison eTable 3. Geriatric Conditions by Hip Fracture TMLD/Gain Groups: Mobility eTable 4. Geriatric Conditions by Hip Fracture TMLD/Gain Groups: Self-care [file jamanetwopen-3-e1919672-s001.pdf]

Supplementary Online Content

Cogan AM, Weaver JA, McHarg M, Leland NE, Davidson L, Mallinson T. Association of length of stay, recovery rate, and therapy time per day with functional outcomes after hip fracture surgery. *JAMA Netw Open*. 2020;3(1):e1919672. doi:10.1001/jamanetworkopen.2019.19672

eTable 1. Hip Fracture Unadjusted Mobility Status at Admission and Discharge, Overall Change, Change per LOS Day, and Length of Stay

eTable 2. Hip Fracture Unadjusted Self-care Status at Admission and Discharge, Overall Change, Change per LOS Day, and Length of Stay

eFigure 1. Definition of Therapy Minutes per LOS Day

eFigure 2. Mobility Gain per Day by TMLD, Inpatient Rehabilitation and Skilled Nursing Facility Comparison

eFigure 3. Self-care Gain per Day by TMLD, Inpatient Rehabilitation and Skilled Nursing Facility Comparison

eTable 3. Geriatric Conditions by Hip Fracture TMLD/Gain Groups: Mobility

eTable 4. Geriatric Conditions by Hip Fracture TMLD/Gain Groups: Self-care

This supplementary material has been provided by the authors to give readers additional information about their work.

© 2020 Cogan AM et al. *JAMA Network Open.*

eTable 1: Hip Fracture Unadjusted Mobility Status at Admission and Discharge, Overall Change, Change per LOS Day, and Length of Stay

		**Mobility Status at Admission** Median [IQR]	**Mobility Status at Discharge** Median [IQR]	**Overall Change in Mobility** Median [IQR]	**Change per LOS Day** Median [IQR]	**LOS** Median [IQR]	**LOS days without PT** Median (%)	**LOS days without OT** Median (%)	**Discharged Home** n (%)
**Low Gain**	**Low TMLD** (n=23)	15.6 [11.4, 17.3]	21.2 [18.5, 22.2]	4.2 [2.1, 7.7]	0.2 [0.1, 0.2]	34.0 [23.0, 50.0]	7.0 (20.6)	12.0 (35.3)	11 (48)
	**Medium TMLD** (n=13)	15.9 [15.0, 16.8]	21.2 [19.1, 23.0]	5.1 [4.1, 6.9]	0.2 [0.2, 0.2]	28.0 [19.0, 31.0]	6.0 (21.4)	6.0 (21.4)	8 (62)
	**High TMLD** (n=13)	15.5 [14.4, 17.2]	19.4 [17.3, 19.6]	3.3 [2.6, 3.7]	0.2 [0.2, 0.2]	18.0 [14.0, 20.0]	3.0 (16.7)	3.0 (16.7)	4 (31)
**Medium. Gain**	**Low TMLD** (n=16)	16.8 [16.0, 17.9]	26.7 [25.1, 28.4]	9.4 [7.3, 13.6]	0.4 [0.3, 0.4]	26.5 [18.0, 35.5]	5.0 (18.9)	7.5 (28.3)	14 (88)
	**Medium TMLD** (n=18)	16.5 [14.8, 17.7]	23.8 [21.8, 25.6]	7.4 [5.2, 9.3]	0.4 [0.3, 0.5]	20.0 [15.0, 23.0]	4.0 (20.0)	4.5 (22.5)	12 (67)
	**High TMLD** (n=17)	17.3 [15.0, 18.3]	23.4 [22.4, 24.3]	6.5 [4.9, 7.6]	0.4 [0.3, 0.5]	16.0 [14.0, 18.0]	3.0 (18.8)	3.0 (18.8)	16 (94)
**High Gain**	**Low TMLD** (n=5)	14.9 [14.4, 19.1]	29.1 [26.7, 30.8]	13.0 [8.6, 14.7]	0.7 [0.6, 0.7]	20.0 [12.0, 22.0]	4.0 (20.0)	4.0 (20.0)	5 (100)
	**Medium TMLD** (n=21)	17.7 [16.6, 19.4]	26.7 [25.8, 30.8]	9.6 [7.5, 12.2]	0.8 [0.7, 1.0]	11.0 [9.0, 16.0]	2.0 (18.2)	2.0 (18.2)	20 (95)
	**High TMLD** (n=24)	17.9 [15.8, 19.2]	27.7 [25.7, 30.8]	11.3 [8.6, 12.8]	0.9 [0.7, 1.2]	13.0 [8.5, 15.0]	1.5 (11.5%)	1.5 (11.5)	18 (75)

IQR: Inter-quartile range

© 2020 Cogan AM et al. *JAMA Network Open.*

eTable 2: Hip Fracture Unadjusted Self-care Status at Admission and Discharge, Overall Change, Change per LOS Day, and Length of Stay

		**Self-care Status at Admission** Median [IQR]	**Self-care Mobility Status at Discharge** Median [IQR]	**Overall Change in Self-care** Median [IQR]	**Change per LOS Day** Median [IQR]	**LOS** Median [IQR]	**LOS days without PT** Median (%)	**LOS days without OT** Median (%)	**Discharged Home** n (%)
**Low Gain**	**Low TMLD** (n=17)	21.4 [20.0, 22.7]	24.1 [20.2, 25.7]	2.1 [0.0, 4.6]	0.1 [0.0, 0.1]	35.0 [22.0, 50.0]	7.0 (20.0)	12.0 (34.3)	7 (41)
	**Medium TMLD** (n=10)	23.3 [22.5, 24.6]	26.6 [24.1, 28.1]	3.0 [1.9, 3.8]	0.2 [0.1, 0.2]	21.0 [16.0, 29.0]	6.0 (28.6)	6.0 28.6)	6 (60)
	**High TMLD** (n=13)	23.1 [20.8, 23.6]	24.6 [22.5, 26.3]	1.7 [0.7, 2.8]	0.1 [0.1, 0.2]	19.0 [14.0, 20.0]	2.0 (10.5)	2.0 (10.5)	3 (23)
**Medium. Gain**	**Low TMLD** (n=22)	23.6 [20.6, 25.2]	32.1 [31.2, 34.0]	8.7 [7.4, 11.4]	0.3 [0.3, 0.4]	25.0 [20.0, 36.0]	5.5 (27.5)	7.5 (30)	18 (82)
	**Medium TMLD** (n=24)	22.3 [20.3, 23.1]	29.2 [26.0, 32.1]	7.3 [4.4, 9.7]	0.3 [0.3, 0.4]	20.0 [17.5, 26.5]	3.5 (17.5)	4.0 (20)	18 (75)
	**High TMLD** (n=22)	23.6 [21.4, 25.2]	29.6 [26.3, 30.3]	5.0 [4.4, 6.2]	0.3 [0.3, 0.4]	16.0 [13.0, 18.0]	2.5 (15.6)	3.0 (18.8)	17 (77)
**High Gain**	**Low TMLD** (n=5)	22.1 [21.4, 23.1]	34.0 [33.0, 35.1]	9.9 [9.8, 13.1]	0.6 [0.6, 0.6]	18.0 [17.0, 23.0]	4.0 (22.2)	5.0 (27.8)	5 (100)
	**Medium TMLD** (n=18)	25.2 [23.1, 25.7]	34.6 [32.1, 36.5]	9.2 [7.3, 12.4]	0.9 [0.7, 1.0]	10.5 [8.0, 14.0]	2.0 (19.0)	2.0 (19.0)	18 (89)
	**High TMLD** (n=19)	24.0 [22.0, 24.6]	33.0 [31.2, 34.0]	8.4 [6.1, 10.4]	0.7 [0.6, 1.0]	11.0 [8.0, 15.0]	3.0 (27.3)	2.0 (18.2)	18 (95)

Note to eTables 1 and 2

© 2020 Cogan AM et al. *JAMA Network Open.*

The median LOS for patients in SNFs (24.0 days; IQR: 17.0, 35.0) was nine days longer than in IRF (15.0 days [IQR: 11.0, 19.0]); however, the median total amount of therapy minutes (IRF: 2070.0 minutes [IQR: 1395.0, 2580.0] ; SNF: 1865.0 minutes [IQR: 1318.0, 2660.0]) and median therapy minutes per LOS day was higher in IRFs (IRF: 135.0 minutes/LOS day [IQR: 126.0, 150.0]; SNF: 77.0 minutes/LOS day [IQR: 65.0, 95.0]).

Median functional mobility status at admission was nearly identical in both SNF (16.57 [IQR: 14.81, 17.92]) and IRF (16.90 [IQR: 15.01, 18.46]) groups.

Self-care Functional self-care scores were similar upon in IRF and SNF settings at admission (IRF: 23.06 [IQR: 21.42, 24.63]; SNF: 23.06 [IQR: 20.84, 25.16]).

© 2020 Cogan AM et al. *JAMA Network Open.*

eFigure 1: Definition of Therapy Minutes per LOS Day

S	S	M	T	W	T	F	S	S	M	T	W	T	F	Total Therapy Time	
			①	②	③	④	⑤	⑥	⑦	⑧	⑨			435	Inpatient Days
				❶	❷	❸	❹		❺	❻				435	Therapy Days

Length of stay = **9 days**
Therapy days = **6 days**
Total therapy minutes = **435 minutes**
Therapy minutes per length of stay day = **435/9 = 48.33**
Therapy minutes per therapy day = **435/6 = 72.5**

© 2020 Cogan AM et al. *JAMA Network Open.*

eFigure 2: Mobility Gain Per Day by TMLD, Inpatient Rehabilitation and Skilled Nursing Facility Comparison

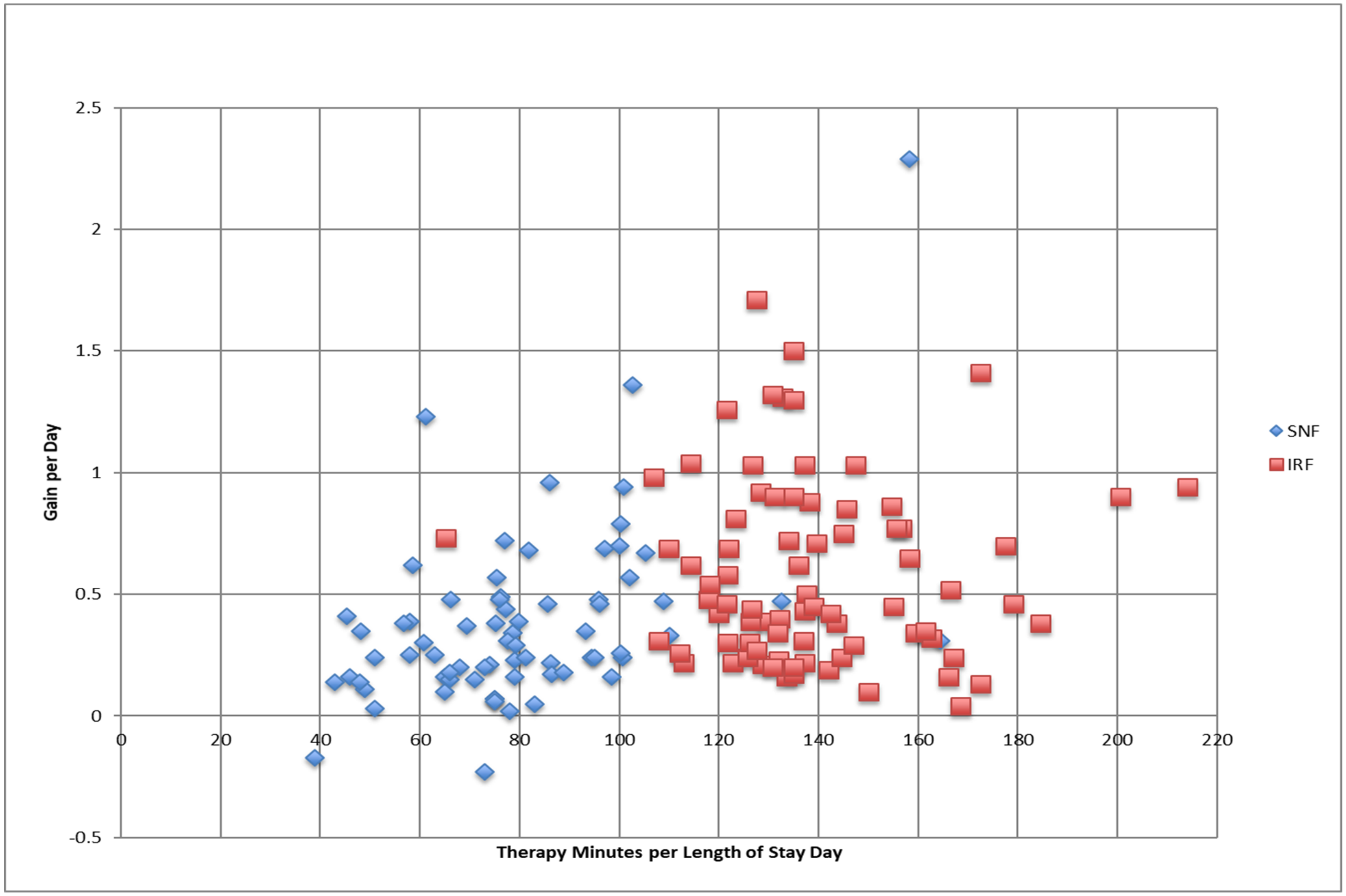

Note: There was wide variation in functional gain per day at all TMLD levels (supplemental file 2A). For example, at 135 therapy minutes per LOS day (median for patients in IRF settings), rate of gain per day ranged from less than 0.25 to more than 1.60 units. At 77 minutes per LOS day (median for patients in SNF settings), gain per day ranged from almost 0 to 1.30 units per day.

© 2020 Cogan AM et al. *JAMA Network Open.*

eFigure 3: Self-care Gain Per Day by TMLD, Inpatient Rehabilitation and Skilled Nursing Facility Comparison

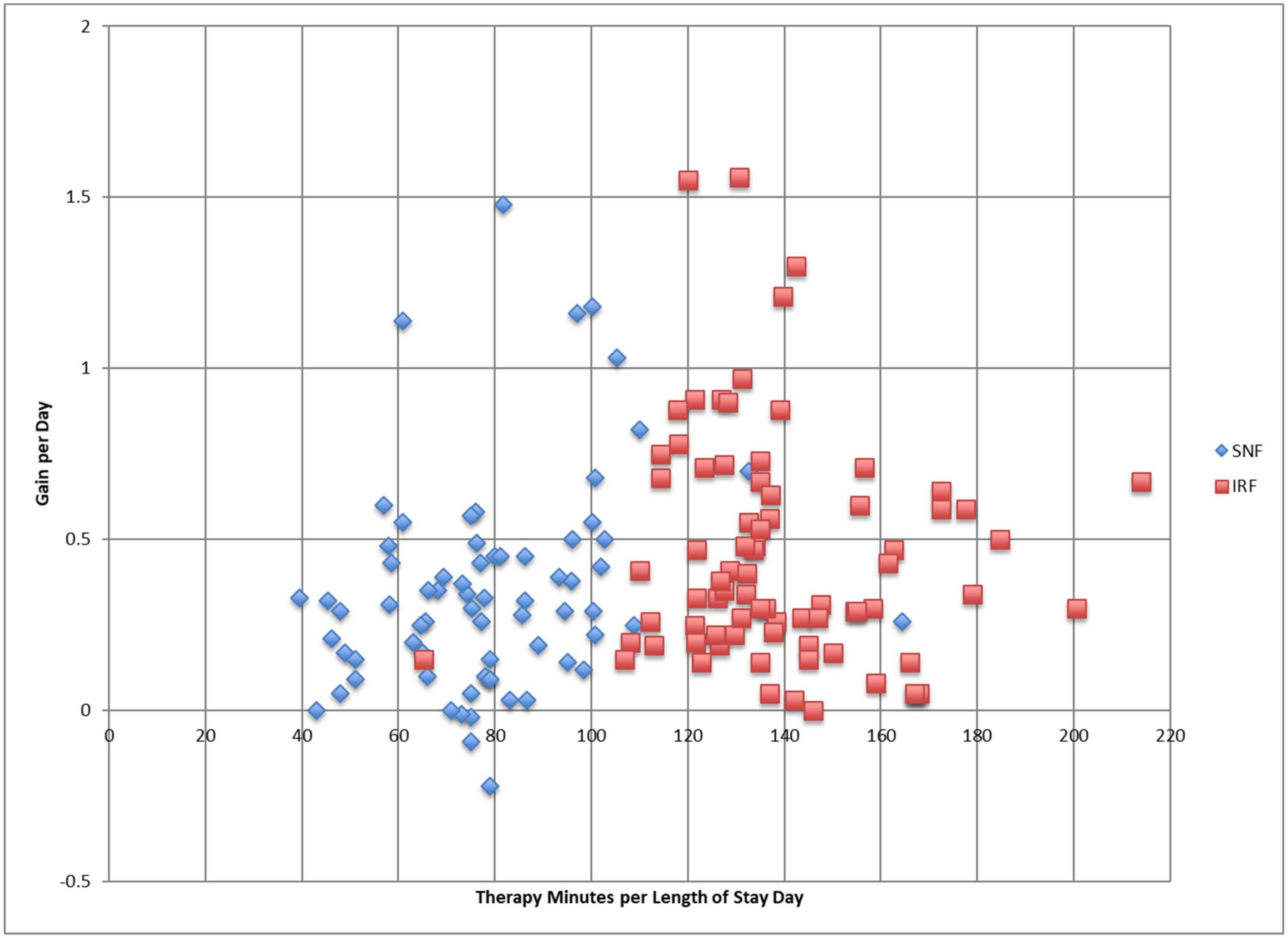

Note: There was substantial variation in self-care gains at all TMLD levels. At 135 therapy minutes per LOS day (median for patients in IRF settings), gain per day ranged from less than 0.20 to 1.50 units per LOS day. At 77 minutes per LOS day (median for patients in SNF settings), gain per day ranged from 0 to 0.60 units per LOS day.

© 2020 Cogan AM et al. *JAMA Network Open.*

eTable 3: Geriatric Conditions by Hip Fracture TMLD/gain groups: Mobility

Subgroup	n	Dementia	Obesity	Falls	New ulcer	Bowel	Bladder	Depression	Vision	Diabetes	Decision making	**3+ conditions**
Low TMLD/ low gain	23	2	4	5	3	6	17	7	7	3	16	14
Low TMLD/ Mediumium gain	16	1	1	1	1	5	9	2	1	3	8	6
Low TMLD/high gain	5	0	2	0	0	0	3	0	0	3	3	2
Medium TMLD/low gain	13	0	0	3	1	1	5	0	3	1	8	3
Medium TMLD/ medium gain	18	0	3	0	0	2	10	3	3	2	9	4
Medium TMLD/ high gain	21	0	3	0	0	1	2	2	3	5	8	2
High TMLD/ low gain	13	0	2	0	1	5	9	4	3	5	4	6
High TMLD/ Medium gain	17	0	0	1	1	2	12	3	7	4	4	6
High TMLD/ high gain	24	0	2	0	0	4	11	1	7	6	4	5

TMLD groups: Low:<80 minutes per LOS day; Medium:80-130 minutes per LOS day; High: >130 minutes per LOS day. Gain groups: Low: <0.25; Medium: 0.25-0.5; High: >0.5.

© 2020 Cogan AM et al. *JAMA Network Open.*

eTable 4: Geriatric Conditions by Hip Fracture TMLD/gain groups: Self-care

Subgroup	n	Dementia	Obesity	Falls	New ulcer	Bowel	Bladder	Depression	Vision	Diabetes	Decision making	**3+ conditions**
Low TMLD/ low gain	17	0	4	4	3	4	11	4	5	3	10	10
Low TMLD/ medium gain	22	3	2	2	1	6	14	4	2	5	14	10
Low TMLD/high gain	5	0	1	0	0	1	4	1	1	1	3	2
Medium TMLD/low gain	10	0	2	2	1	1	4	1	1	2	4	3
Medium TMLD/ medium gain	24	0	1	1	0	2	12	3	6	3	15	5
Medium TMLD/ high gain	18	0	3	0	0	1	1	1	2	3	6	1
High TMLD/ low gain	13	0	3	1	1	5	8	5	5	7	3	8
High TMLD/ Medium gain	22	0	1	0	1	5	16	1	8	6	5	7
High TMLD/ high gain	19	0	0	0	0	1	8	2	4	2	4	2

TMLD groups: Low:<80 minutes per LOS day; Medium:80-130 minutes per LOS day; High: >130 minutes per LOS day. Gain groups: Low: <0.2; Medium: 0.2-0.5; High: >0.5.

© 2020 Cogan AM et al. *JAMA Network Open.*
